# A new approach to predicting shoulder dystocia: fetal clavicle measurement

**DOI:** 10.3906/sag-2011-145

**Published:** 2021-08-30

**Authors:** Elif TERZİ

**Affiliations:** 1 Department of Gynecology and Obstetrics, Faculty of Medicine, Lokman Hekim University, Ankara Turkey

**Keywords:** Clavicle/growth and development, fetal macrosomia, shoulder dystocia, parturition

## Abstract

**Background/aim:**

This study aimed to evaluate the relationship between second- and third-trimester clavicle lengths and birth weight and shoulder dystocia.

**Materials and methods:**

This prospective observational study included 181 patients who presented to the Private Etlik Lokman Hekim Hospital for routine pregnancy visits between March 2019 and March 2020. In addition to routine pregnancy examinations, the patients also underwent ultrasonography twice at weeks 20–23 and 33–36 to determine the length of the fetal clavicle. The patients were evaluated for shoulder dystocia in the second stage of labor. The birth weight of the neonates was recorded. The primary objective of this study was to establish the relationship between third-trimester clavicle length and shoulder dystocia.

**Results:**

Fetal clavicle length increases in the second trimester with the advancing gestational week but does not significantly change in the third trimester. We did not observe any significant difference for second trimester clavicle length between the type of delivery, birth weight, or shoulder dystocia. Moreover, we did not observe any significant difference for third trimester clavicle length between types of delivery. However, we found a significant relationship between third trimester clavicle length and birth weight and shoulder dystocia. The median third-trimester clavicle length was 39.5 mm (range: 30.7–43.9) in neonates who did not develop shoulder dystocia and 42.5 mm (range: 41.4–43.1) in the 3 neonates who developed shoulder dystocia. The third-trimester clavicle length cut-off for shoulder dystocia was calculated as 41.35 mm (sensitivity: 100.00%, specificity: 83.82%, accuracy: 84.5%). The third-trimester clavicle length cut-off for macrosomia (defined as birth weight of ≥4100 g) was 40.75 mm (sensitivity: 87.50%, specificity: 77.56%, accuracy: 78.05%).

**Conclusion:**

Third-trimester fetal clavicle length, an important component of biacromial diameter, as measured by ultrasonography, is a practical and significant method for predicting macrosomia and shoulder dystocia.

## 1. Introduction

Accurate estimation of birth weight (BW) is important for predicting and preventing birth complications. However, this issue is still an unresolved problem in obstetrics [1]. Macrosomia, defined as excessive weight at birth, is a serious risk factor for a complicated delivery [2]. No single definition is currently universally accepted, although macrosomia is commonly defined as birth weight of >4000–4500 g or >90th percentile [3]. Fetal weight is influenced by many genetic and environmental factors [4], including maternal weight before pregnancy [2], weight gain during pregnancy [5], parity [6], and fetal sex [7]. Therefore, the best time to determine estimated fetal weight (EFW) is the last trimester of pregnancy [8]. Numerous risk factors have been described for macrosomia, combinations of which are present in most macrosomic neonates [9]. It is essential to detect macrosomia in the antenatal period to adequately plan for any potential complications and needs that may arise during birth and neonatal care [10]. Macrosomia is associated with increased cesarean section rates and shoulder dystocia, prolonged hospitalization [11], and even stillbirth [12]. There are also studies indicating that macrosomia is not significantly associated with shoulder dystocia [13].

Routine ultrasonography (US) examinations in pregnancy that monitor intrauterine development and fetal growth are used to estimate fetal weight [14]. EFW is calculated using certain formulas that aim to accurately predict BW [15]. There are numerous antenatal fetal weight estimation methods. Measurements are combined with nonlinear regression analysis or volumetric methods to develop formulas to estimate fetal weight. A 2010 study investigated the sensitivity of 36 different EFW measurement tools and found that none of the methods were superior, and false positives were significantly more common when BW was >4500 g [16]. Moreover, it was concluded that 3D US, which is more sensitive for volumetric measurements, was not more sensitive than 2D US in estimating fetal weight [17].

Fetal growth is variable; therefore, researchers proposed customized fetal growth curves to replace those presented by the World Health Organization for the diagnosis of macrosomia in antenatal follow-up. However, these customized growth curves were not found to reduce the risk of C-section secondary to intrapartum dystocia [18].

One obstetrical emergency of medicolegal significance associated with macrosomia is shoulder dystocia [19]. The most objective definition of shoulder dystocia is a head-to-body time interval of ≥60 s or requiring ancillary obstetric maneuvers. According to this definition, the incidence of shoulder dystocia is 10% [20]. A different definition indicates that shoulder dystocia is diagnosed when the contraction that follows the emergence of the fetal head is not sufficient for the delivery of the shoulders. Using this definition, the incidence of shoulder dystocia is approximately 2%–3% [21].

Shoulder dystocia is still a challenge in clinical practice due to not being predictable. Current knowledge holds that increased fetal weight is associated with an increased risk of shoulder dystocia. However, dystocia can occur not only in macrosomic but also nonmacrosomic fetuses [22]. For this reason, researchers are seeking new antenatal parameters to predict shoulder dystocia [1,23].

In the present study, we investigated the diagnostic value of fetal clavicle length in predicting macrosomia and shoulder dystocia.

## 2. Materials and methods

This prospective observational study included patients who presented to the Private Etlik Lokman Hekim Hospital Obstetrics Clinic between March 2019 and March 2020. 

After approval by the local ethics committee (date: 20/03/2019, number: 2019/11-2019008, Lokman Hekim University ethics committee), singleton pregnancies between 20 and 23 weeks without fetal abnormalities were included in the study regardless of parity or previous type of delivery. Mothers with type 1 or type 2 diabetes were excluded. All subjects underwent a 75-g oral glucose tolerance test, and one patient was excluded due to being diagnosed with gestational diabetes. The purpose and method of the study were explained to the participants and written consent was obtained.

The gestational age of the fetus was confirmed by comparing results to first-trimester US measurements. All participants had adequate amniotic fluid and none of the participants were in active labor. Gestational age was rounded off to the nearest lower week for days ≤4 and the nearest higher week for days ≥5. Fetal clavicle length was measured using US in order to evaluate fetal anatomy in the second trimester and to monitor fetal growth in the third trimester. All ultrasound measurements were made by a single diagnostic medical sonographer, Nurten ÇETİN MD, using a Siemens (Germany) ACUSON X700 with a curved linear array 4–12 MHz ultrasound transducer. EFW was calculated using the Hadlock formula based on biparietal diameter (BPD), head circumference (HC), abdominal circumference (AC), and femur length (FL). During the second-trimester axial ultrasound, the clavicle was determined by its characteristically curvy structure. Electronic calipers were placed on the lateral and medial sides of the clavicle, respectively. The measurement was repeated during the third trimester while the head was in the occiput transverse position, and the clavicle was located and measured. For each examination, three individual images of the fetal clavicle were obtained. The measurements were in accordance with the method described by Yarkoni and Sherer [24,25].

The study initially included 181 patients. After the first clavicle measurement, 13 subjects were excluded due to intrauterine death (1), preterm birth (3), gestational diabetes mellitus (1), or changing hospitals (8), and the second measurement was performed for 168 patients. Of those patients, 164 gave birth in our hospital. Patients who did not give birth in our hospital were excluded from macrosomia and shoulder dystocia analyses; however, their data from the first and second measurements were included in analysis to determine clavicle length change. Type of delivery and the development of shoulder dystocia were recorded. Shoulder dystocia was diagnosed when the contraction that followed the emergence of the fetal head was not sufficient for the delivery of the shoulders. In 1 dystocia patient, the McRoberts maneuver was sufficient to achieve delivery [26]. In the remaining 2 dystocia patients, delivery was achieved after performing the McRoberts maneuver followed by the anterior Rubin maneuver [27]. All neonates were delivered without any complications, including clavicle fracture or brachial plexus paralysis. 

Within the first 1 h after birth, all neonates were examined by a pediatrician and evaluated as healthy. Neonatal weight was measured by a neonatal nurse using a digital scale. Neonates with BW of ≥4100 g were diagnosed with macrosomia.

The primary objective of the study was to establish the relationship between third-trimester clavicle length and shoulder dystocia. The secondary objective was to establish the relationship between third-trimester clavicle length and fetal macrosomia.

### 2.1. Statistical analysis

Normality of the distribution of numerical data was tested using the Shapiro–Wilk test. Numerical variables were expressed as mean ± standard deviation and median (minimum–maximum), and categorical variables were expressed as numbers (n) and percentages. For numerical data, the independent samples t-test and the Mann–Whitney U-test were used for pairwise comparison, and the Kruskal–Wallis test was used for the comparison of three or more groups. If the result was statistically significant, the Dunn–Bonferroni correction was applied. The correlations between variables were investigated using the Pearson and Spearman rho correlation coefficients. Third-trimester fetal clavicle length and birth weight were hypothesized to be clinically relevant for shoulder dystocia, and this relationship was examined using both direct and partial correlation coefficients.

Receiver operating characteristic (ROC) curve analysis was performed to determine the third-trimester fetal clavicle length cut-off points for shoulder dystocia and BW of ≥4100 g. Areas under the curve (AUCs) and 95% confidence intervals were calculated. If the AUC was >0.50, sensitivity and specificity were calculated.

Data were analyzed using IBM SPSS version 21.0 for Windows (released 2012; IBM Corp., Armonk, NY, USA). Values of p < 0.05 were accepted as statistically significant.

## 3. Results

Demographic characteristics of patients are given in Table 1. Among the participants, 47% were primiparous and 53% multiparous. While 56.6% of the participants gave birth by cesarean delivery (CD), 43.4% had vaginal delivery (VD). The indications for patients with cesarean delivery are as follows: previous cesarean section (38), malpresentation (18), cephalopelvic disproportion (18), fetal distress (5), placenta previa (5), preeclampsia (4), oligohydramnios (2), prolonged first stage (2), and macrosomia (1). The mean second-trimester clavicle length was 25.17 ± 1.69 mm and the mean third trimester clavicle length was 38.77 ± 3.00 mm. There was no significant difference between vaginal and abdominal delivery for the parameters of second-trimester clavicle length (25.21 ± 1.61 vs 25.06 ± 1.90, p = 0.64) and third-trimester clavicle length (39.03 ± 2.94 vs 39.06 ± 2.41, p = 0.58). A statistically significant correlation was found between estimated fetal weight and birth weight (n = 164 rho = 0.865; p < 0.001). There were 8 patients who delivered neonates larger than 4100 g.

**Table 1:** Demographic and clinic characteristics of patients.

Age (years) (n = 181)		Second trimester clavicle length (mm)	
mean ± SD	31.43 ± 4.66	mean ± SD	25.17 ± 1.69
median (min; max)	31 (20; 48)	median (min; max)	25.2 (21.4;29.7)
Initial BMI (kg/m2) (n = 181)		Third trimester clavicle length (mm)	
mean ± SD	24.03 ± 3.79	mean ± SD	38.77 ± 3.00
median (min; max)	23.7 (16; 37)	median (min; max)	39.5 (30.7; 43.9)
Final BMI (kg/m2) (n = 164)		Newborn weigth (g) (n = 164)	
mean ± SD	29.52 ± 4.25	mean ± SD	3316.43 ± 432.32
median (min; max)	28.75 (21; 42)	median (min; max)	3305 (2235; 4495)
Gravidity (n = 181)		Estimated fetal weigth (g) (n= 164)	
primigravida	85 (47.0)	mean ± SD	3340.54 ± 405.5
multigravida	96 (53.0)	median (min; max)	3390 (2300; 4300)
Parity (n = 181)		AC (mm) (n = 164)	
primiparous	73 (40.3)	mean ± SD	345.26±25.28
multiparous	108 (59.7)	median (min; max)	348 (284; 396)
Abortion (n = 41)		APGAR score 1 min (n = 164)	
1	28 (68.3)	median (min; max)	8 (3; 9)
2+	13 (31.7)	APGAR score 5 min (n = 164)	
Macrosomia history	n (%)	median (min; max)	10 (6; 10)
no	166 (91.7)	Sex (n = 164)	n (%)
yes	15 (8.3)	female	84 (51.2)
Week in delivery (n = 164)		male	80 (48.8)
mean ± SD	38.76 ± 1.15		
median (min; max)	39 (34; 41)		

Second-trimester clavicle length increased with gestation. Third-trimester clavicle length was not significantly correlated with weeks of gestation. Moreover, second- and third-trimester clavicle lengths were not significantly correlated (p = 0.589, rho = 0.042).

Clavicle measurement results by week of gestation are given in Table 2.

**Table 2:** Clavicle measurement results by week of gestation.

Gestational week	n	mean ± SD	median (min; max)	p
20	9	23.01 ± 0.98	22.9 (21.8; 25.0)	c2 = 45.133; p < 0.001
21	50	24.3 ± 1.27	24.4 (21.8; 27.1)	
22	91	25.47 ± 1.56	25.5 (21.4; 29.3)	
23	31	26.33 ± 1.62	26.4 (22.8; 29.7)	
33	10	37.92 ± 4.02	39.4 (32.7; 43.1)	c2 = 0.373; p = 0.946
34	98	38.74 ± 2.95	39.4 (30.7; 43.1)	
35	45	39.01 ± 2.97	39.9 (30.9; 43.9)	
36	15	38.80 ± 2.83	39.3 (32.2; 42.7)	

c2: Kruskal–Wallis test

The mean BW of this study subjects was 3316 g (3354 g for the VD group and 3286 g for the CD group, p = 0.391). Mean birth weight was significantly higher in infants with shoulder dystocia (4276 g) than in infants without shoulder dystocia (3320 g) (p < 0.001).

Second-trimester clavicle length did not show significant difference between patients who experienced shoulder dystocia and those who did not (24.60 ± 1.51 vs 25.25 ± 1.61, p = 0.434). On the other hand, this measurement was significantly positively but negligibly correlated with birth weight (p = 0.021, r = 0.180). These results are given in Table 3. 

**Table 3:** Second-trimester and third-trimester clavicle lengths and associated variables.

	Second-trimester clavicle length				Third-trimester clavicle length		
Neonatal birth weight range (n = 164)	n	mean ± SD	median(min; max)	p	mean ± SD	median (min; max)	p
<2700	9	25.20 ± 1.70	25.0 (22.9; 27.7)	c2 = 11.755;p = 0.228	36.97 ± 3.22	37.6 (32.1; 41.4)a	c2 = 35.033;p < 0.001
2701–2900	17	25.22 ± 1.85	25.6 (21.8; 28.5)		37.96 ± 2.36	38.7 (32.0; 40.8) b	
2901–3100	25	24.59 ± 1.25	25.0 (21.4; 26.9)		37.51 ± 3.13	38.2 (30.9; 42.9) c,d,e	
3101–3300	31	25.07 ± 1.42	25.2 (21.9; 27.4)		38.37 ± 2.94	39.2 (32.0; 43.9)	
3301–3500	31	25.01 ± 1.38	24.8 (22.4; 27.9)		39.86 ± 2.47	40.3 (32.2; 42.8) c	
3501–3700	25	25.69 ± 1.79	25.8 (21.9; 28.6)		39.04 ± 2.91	39.5 (31.2; 42.8)	
3701–3900	15	25.99 ± 2.32	26.2 (23.2; 29.3)		40.47 ± 1.23	40.7 (38.0; 42.2) d	
3901–4100	3	25.00 ± 1.44	24.6 (23.8; 26.6)		37.53 ± 6.01	39.9 (30.7; 42.0)	
4101–4300	4	24.50 ± 1.79	24.5 (22.9; 26.1)		39.38 ± 4.94	41.5 (32.0; 42.5)	
4301–4500	4	27.18 ± 2.00	27.1 (24.8; 29.7)		42.30 ± 1.09	42.7 (40.8; 43.1) a,b,e	
Delivery type (n = 164)							
vaginal	71	25.21 ± 1.61	25.3 (21.9; 28.5)	t = 0.468;p = 0.641	39.03 ± 2.94	40.0 (30.7; 43.1)	Z = 0.544;p = 0.586
cesarean	93	25.06 ± 1.90	24.6 (21.4; 29.7)		39.06 ± 2.41	39.7 (32.1; 42.7)	
Shoulder dystocia(n = 71)							
no	68	25.25 ± 1.61	25.3 (21.9;28.5)	Z = 0.829; p = 0.434	38.92 ± 2.91	39.9 (30.7; 42.8)	Z = 2.530; p = 0.004
yes	3	24.60 ± 1.51	24.8 (23.0; 26.0)		42.33 ± 0.86	42.5 (41.4; 43.1)	

c2:Kruskal–Wallis test, t: independent sample t test, Z: Mann–Whitney U test, Adjusted p-values Obtained for Bonferroni–Dunn a : 0.027, b :0.027, c :0.043, d :0.042, e :0.020

Third-trimester clavicle length showed significant difference between patients who experienced shoulder dystocia and those who did not (42.33 ± 0.86 vs 38.92 ± 2.91, p = 0.004). The mean third-trimester clavicle length was 39.9 mm (range: 30.7–42.8) in neonates who did not develop shoulder dystocia and 42.5 mm (range: 41.4–43.1) in the 3 neonates who did develop shoulder dystocia (Table 3).

According to the ROC analysis, the third-trimester clavicle length cut-off for shoulder dystocia was 41.35 mm (AUC: 0.934, SE: 0.044, sensitivity: 100.00%, specificity: 83.82%, accuracy: 84.5%; p = 0.011) (Table 4). The ROC curve is shown in Figure a.

**Table 4:** Predictive value of third-trimester fetal clavicle length for prediction of shoulder dystocia and fetal macrosomia.

	AUC(Std Error)	95%CI for AUC	p	Cut-off	Sensitivity(%)	Specificity(%)	PPV	NPV	Accuracy
Third-trimester fetal clavicle length for prediction of shoulder dystocia	0.934 (0.044)	0.847–1.000	0.011	≥41.35	100.00	83.82	21.43	100.00	84.51
Third-trimester fetal clavicle length for prediction of fetal macrosomia	0.807 (0.108)	0.596–1.000	0.003	≥40.75	87.50	77.56	16.67	99.18	78.05

**Figure a:**
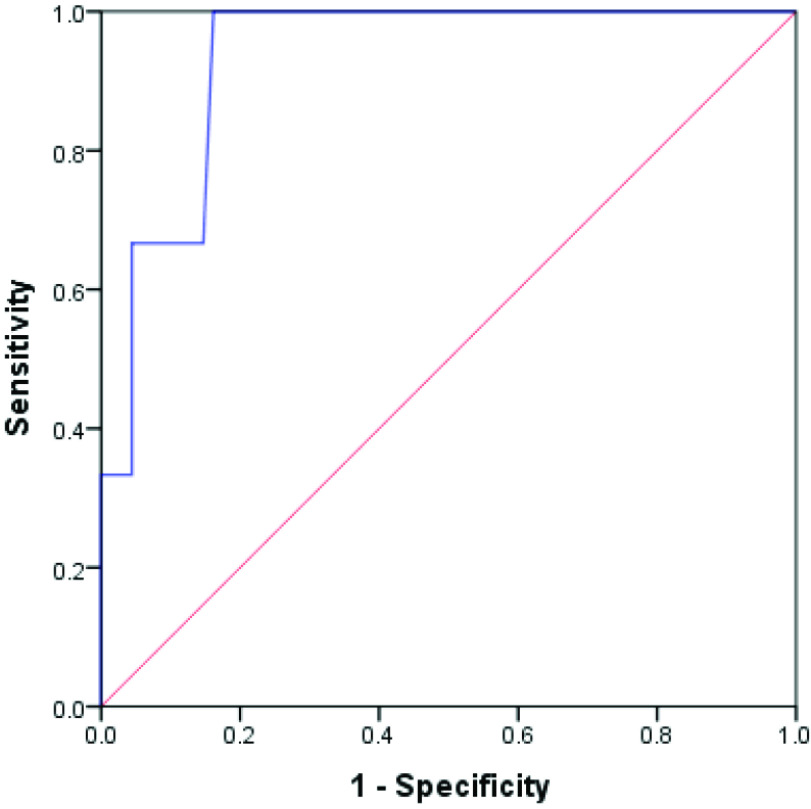
ROC curve for third-trimester fetal clavicle length for prediction of shoulder dystocia.

**Figure b:**
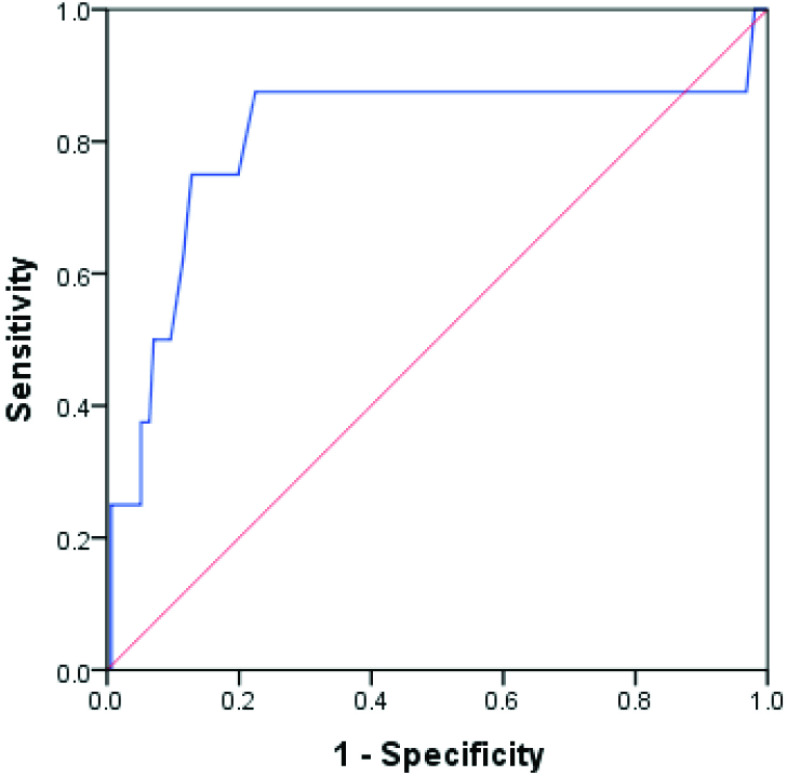
ROC curve for third-trimester fetal clavicle length for prediction of macrosomia.

According to the ROC analysis, the third-trimester clavicle length cut-off for macrosomia (defined as BW of ≥4100 g) was 40.75 mm (AUC: 0.807, SE: 0.108, sensitivity: 87.50%, specificity: 77.56%, accuracy: 78.05%; p = 0.003) (Table 4). The ROC curve is shown in Figure b.

## 4. Discussion

In this study, the relation of clavicle length with macrosomia and shoulder dystocia was evaluated. In the patients included in the study, fetal clavicle length measurement was performed at the third trimester, and the presence of shoulder dystocia and macrosomia in the deliveries of the patients were evaluated. As a result of the study, a statistically significant relationship was shown between the measurement of the third-trimester clavicle length and macrosomia (p = 0.003). Likewise, the success of this measurement in predicting shoulder dystocia was found to be statistically significant (p = 0.011). 

Routine antenatal care now includes US examinations to estimate fetal weight and especially to determine fetal macrosomia. A 2013 Turkish study reported the incidence of macrosomia to be 5.9% for mothers without gestational diabetes [28]. In this study, 4.88% of the neonates had a BW of 4100 g or above.

Numerous studies have used conventional fetal measurements such as BPD, FL, AC, and HC to estimate fetal weight. Unfortunately, these estimations become increasingly inaccurate with increasing actual weight [16], leading to a search for other more accurate antenatal parameters.

A 2016 study estimated fetal weight using a formula based on femur length and soft tissue thickness of the middle thigh. However, the authors found a weak correlation between EFW and actual birth weight [1]. 

A different study investigated the utility of front-abdominal wall thickness in estimating fetal weight and reported that the accuracy of this parameter in predicting macrosomia was similar to those of AC and EFW [23].

Antenatal diagnosis of macrosomia is crucial to predict and prepare for feto-maternal complications and legal implications, including shoulder dystocia [19]. Although shoulder dystocia can also occur in nonmacrosomic fetuses, it increases with increased fetal weight and complicates about 3% of births [21]. In this study, the incidence of shoulder dystocia was 4.2%. Previous studies have shown that the risk of shoulder dystocia increases with increasing fetal weight [22]. Consistently with the literature, in this study, the mean BW of infants with shoulder dystocia was 4276 g compared to 3323 g in infants without shoulder dystocia (p < 0.001).

Anthropometric assessments indicate a significantly larger shoulder circumference in pregnancies complicated with shoulder dystocia [29]. However, it is difficult to measure shoulder circumference by US. Instead, fetal biacromial diameter (BAD) is used to estimate shoulder circumference. Since BAD cannot be calculated from a single US image, this value is calculated using measurements from multiple US images.

In 1997, Winn et al. compared intrauterine and postpartum measurements to investigate the relationship between fetal measurements and neonatal BAD. Accordingly, they stated that the best predictor for neonatal BAD is intrauterine fetal chest circumference followed by arm circumference [30].

In 2019, Youssef et al. proposed a novel and simple method to predict fetal macrosomia and shoulder dystocia. Using ultrasound measurements, they calculated fetal BAD by adding the transverse thoracic diameter and 2 times the upper-arm length. They demonstrated that AC and BAD were similar in their ability to estimate fetal weight [31].

BAD is the distance between the acromial processes of the scapulae, which join the clavicle at the acromioclavicular joint [32]. Hence, it may be more practical to measure the clavicle to calculate BAD. However, the available literature on intrauterine measurements of clavicle length, a key component of BAD, only consists of nomogram studies.

The first study to use US to measure the clavicle was conducted by Yarkoni et al. in 1985 [24]. They found a simple relationship between clavicular length and gestational age, and stated that gestational age (in weeks) was approximately equal to the length of the clavicle (in millimeters). This study reported that clavicle measurement could be useful for estimating gestational age and to detect congenital anomalies affecting the clavicle (such as cleidocranial dysostosis and Holt–Oram syndrome).

A 2006 study by Sherer et al. that included 623 pregnant women demonstrated that the “1 mm–1 week” rule described by Yarkoni could be off by 6 weeks and that the nomogram needed to be revised [25]. We did not aim to establish a nomogram in this study, but like Sherer et al., we observed that the relationship between gestational week and clavicle length did not follow the “1 mm–1 week” rule. In this study, the mean clavicle length was 23 mm at week 20, 24 mm at week 21, 25 mm at week 22, and 26 mm at week 23. In the last trimester, the mean clavicle length was approximately 39 mm at weeks 33 through 36. These study results do not indicate that gestational week matches clavicle length in millimeters; however, clavicle length did increase with gestational age in the second trimester.

Both studies noted that clavicle length can be useful for determining gestational age and diagnosing abnormalities affecting the clavicle, as well as for determining macrosomia, a risk factor for shoulder dystocia.

A 2017 systematic review by Maruotti et al. evaluated a total of 287 pregnant women from 3 studies for macrosomia, aiming to predict macrosomia using third-trimester fetal abdominal and thigh soft tissue thickness by US. They calculated the AUC value of fetal soft tissue thickness for macrosomia prediction as 0.92, sensitivity as 80%, specificity as 95%, and accuracy as 80% [33].

Youssef et al. investigated the utility of fetal BAD in predicting macrosomia among term pregnant women and calculated the BAD cut-off as 15.4 cm (AUC: 0.987) [31]. They also obtained higher sensitivity (96.4%) and specificity (97.14%) values compared to Maruotti et al. [33]. In this study, the third-trimester clavicle length cut-off for macrosomia (defined as BW of ≥4100 g) was 40.75 mm, AUC: 0.807, sensitivity: 87.50%, specificity: 77.56%, accuracy: 78.05% (p = 0.003).

Duryea et al. calculated the femur length-to-abdominal circumference (FL/AC) cut-off for predicting shoulder dystocia as 0.20, AUC as 0.70, sensitivity as 63.6%, and specificity as 69.9% [34].

Youssef et al. investigated the utility of fetal BAD in predicting shoulder dystocia and calculated the BAD cut-off as 15.4 cm, AUC as 0.939, sensitivity as 95%, specificity as 86%, and accuracy as 86.7% [31].

In this study, third-trimester clavicle length could significantly predict shoulder dystocia. According to the ROC analysis, the third-trimester clavicle length cut-off for shoulder dystocia was 41.35 mm, AUC was 0.934, SE was 0.044, sensitivity was 100.00%, specificity was 83.82%, and accuracy was 84.5% (p = 0.011).

Among these study subjects, in the VD group (n = 71), third-trimester clavicle length was ≥41.35 mm in all 4 neonates with a BW of ≥4100 g, 3 of whom developed shoulder dystocia. Similarly, third-trimester clavicle length was ≥41.35 mm in 28 out of 71 vaginally delivered neonates. Four of these 28 neonates had a BW of ≥4100 g, 3 of whom developed shoulder dystocia. These statistical data are particularly important in that they demonstrate that clavicle length alone cannot predict the risk of shoulder dystocia but is significant when evaluated together with macrosomia. 

The strength of this study is that it was a prospective observational study. The limitation of this study is its small sample size. However, the AUC value was statistically significant, and the specificity and sensitivity values were considerably high. To the best of our knowledge, this study is the first to investigate the correlation between fetal clavicle length and macrosomia and shoulder dystocia. Using the data obtained by a single radiologist may also be an advantage of this study. Further studies with larger samples are needed. Comparisons of clavicle length with other fetal parameters are among the studies that might be conducted for the antenatal detection of macrosomia. 
